# Comparative analysis of chemical similarity methods for modular natural products with a hypothetical structure enumeration algorithm

**DOI:** 10.1186/s13321-017-0234-y

**Published:** 2017-08-16

**Authors:** Michael A. Skinnider, Chris A. Dejong, Brian C. Franczak, Paul D. McNicholas, Nathan A. Magarvey

**Affiliations:** 1 Department of Biochemistry and Biomedical Sciences, Michael G. DeGroote Institute for Infectious Disease Research, McMaster University, Hamilton, ON Canada; 2 Department of Chemistry and Chemical Biology, Michael G. DeGroote Institute for Infectious Disease Research, McMaster University, Hamilton, ON Canada; 3 Department of Mathematics and Statistics, McMaster University, Hamilton, ON Canada; 4 Department of Mathematics and Statistics, MacEwan University, Edmonton, AB Canada

**Keywords:** Chemical similarity, Natural products, Chemical fingerprints, Chemical structure enumeration

## Abstract

Natural products represent a prominent source of pharmaceutically and industrially important agents. Calculating the chemical similarity of two molecules is a central task in cheminformatics, with applications at multiple stages of the drug discovery pipeline. Quantifying the similarity of natural products is a particularly important problem, as the biological activities of these molecules have been extensively optimized by natural selection. The large and structurally complex scaffolds of natural products distinguish their physical and chemical properties from those of synthetic compounds. However, no analysis of the performance of existing methods for molecular similarity calculation specific to natural products has been reported to date. Here, we present LEMONS, an algorithm for the enumeration of hypothetical modular natural product structures. We leverage this algorithm to conduct a comparative analysis of molecular similarity methods within the unique chemical space occupied by modular natural products using controlled synthetic data, and comprehensively investigate the impact of diverse biosynthetic parameters on similarity search. We additionally investigate a recently described algorithm for natural product retrobiosynthesis and alignment, and find that when rule-based retrobiosynthesis can be applied, this approach outperforms conventional two-dimensional fingerprints, suggesting it may represent a valuable approach for the targeted exploration of natural product chemical space and microbial genome mining. Our open-source algorithm is an extensible method of enumerating hypothetical natural product structures with diverse potential applications in bioinformatics.

**Electronic supplementary material:**

The online version of this article (doi:10.1186/s13321-017-0234-y) contains supplementary material, which is available to authorized users.

## Background

Quantifying the molecular similarity of chemical structures is a central task in cheminformatics [1, 2]. The assumption that similar molecules are more likely to have similar biological or physicochemical properties than dissimilar ones [3] underlies the diverse applications of molecular similarity calculations in drug discovery, particularly in ligand-based virtual screening and medicinal chemistry, but also in toxicology, chemogenomics, and pharmacology. Consequently, a large and diverse set of methods for the efficient abstract representation of chemical information is available. Although both one-dimensional and three-dimensional descriptors have been developed, two-dimensional molecular fingerprint algorithms, which decompose a chemical graph into a sequence of bits, remain the most common method for representing structural information in the context of assessing molecular similarity [4]. Once generated, chemical fingerprints can be rapidly compared to one another with the widely used Tanimoto coefficient, or one of several other distance metrics [5], in order to quantify the similarity of any two chemical structures.

A number of publicly available datasets [6–8] have been developed for use in benchmarking studies, and several groups have compared the performance of existing two-dimensional chemical fingerprint algorithms on these benchmark datasets [9–12]. A recent review concluded that, while commonly used fingerprinting algorithms have similar performances, circular fingerprints generally perform best [4]. Several studies have also concluded that two-dimensional similarity search methods outperform three-dimensional methods [12]. A number of groups have additionally investigated the performance of several distance (or similarity) metrics [5, 13–15], or combinations thereof [16–18], used to compare chemical fingerprints. These results generally validate the popularity of the Tanimoto coefficient.

Despite these extensive analyses of the performance of molecular descriptors and distance metrics, no comparative analysis of the performance of chemical fingerprinting algorithms on the unique and diverse scaffolds of natural products has to date been reported. Natural products and their derivatives represent a historically invaluable source of industrial and pharmaceutical agents, and the basis for the majority of approved small molecule clinical drugs [19]. These complex small molecules are biosynthesized from simple metabolic building blocks by large, multi-domain enzymes or enzyme complexes in combinatorial strategies [20]. Quantifying chemical similarity is therefore a particularly important task for natural products due to their potent biological activities. In particular, the ability to cheminformatically determine whether a molecule is a member of a known class of bioactive natural products—for instance, the glycopeptides or β-lactams—may facilitate the targeted exploration of chemical space. Moreover, the ability to reliably associate putative natural product structures generated by genomic structure prediction with known natural product classes could facilitate the targeted mining of microbial genomes.

The large and structurally complex scaffolds of natural products distinguish them from synthetic agents. In particular, cheminformatic studies have reported that natural products have greater chemical diversity, greater molecular weight, greater three-dimensional complexity (including more rotatable bonds, more stereocenters, and a higher fraction of sp^3^ carbons), lower hydrophobicity and greater polarity, fewer aromatic rings, more heteroatoms, and more hydrogen bond donors and acceptors relative to synthetic agents [21, 22], and contain unique pharmacophores and ring systems [23, 24]. However, existing collections are biased towards synthetically tractable scaffolds [25] and by Lipinski’s ‘rule of 5’ physicochemical parameters for orally bioavailable drugs [26], despite the fact that a significant proportion of approved natural product drugs violate these rules [27]. A recent analysis reported that only 17% of natural product ring scaffolds are present in commercially available screening collections [24]. Because of these disparities between naturally occurring and commercially available chemistries, several new methods to chart natural product chemical space have been described [28–32]. However, in the context of new algorithm development to explore natural product chemical space, a rigorous assessment of existing methods for quantifying the molecular similarity of natural products is essential.

**Fig. 1:**
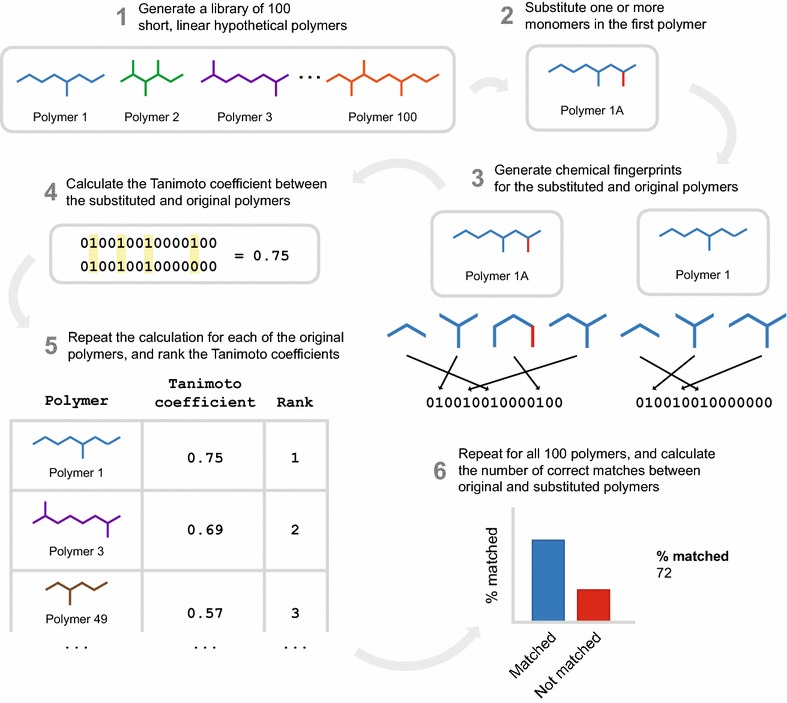
Application of an algorithm for hypothetical modular natural product structure enumeration to comparative analysis of chemical similarity methods. A short, linear biomimetic polymer is generated by LEMONS, and one or more monomers are substituted. Tailoring reactions may be executed on the original and/or modified polymers. The modified polymer is compared to the entire original library with one of 18 chemical similarity algorithms. A correct match is scored if the modified structure displays greater chemical similarity to the original structure than to any of the other structures within the library. The process is repeated for each original polymer and the fraction of correct matches is calculated. Each experiment is repeated 100 times

In order to investigate the performance of molecular similarity algorithms on natural product structures, we developed LEMONS (Library for the Enumeration of MOdular Natural Structures). LEMONS is a software package designed to enumerate hypothetical modular natural product structures, modify their monomer composition or tailoring reactions, and compare the original and modified structures using two-dimensional molecular fingerprints (Fig. 1, Methods). This method allows the definition of a true match between the original and modified scaffolds originating from the same in silico assembly line. Consequently, the proportion of correct matches between original and modified structures can be derived for each fingerprint. In biosynthetic terms, this proportion can be thought to represent the ability of a similarity search method to identify modular natural products arising from the same in silico enzymatic assembly line. We apply the LEMONS algorithm to investigate the performance of 17 chemical fingerprints with respect to modular natural product structures, including linear and cyclic nonribosomal peptides, polyketides, and hybrids, with a diverse range of tailoring reactions and starter units. We additionally investigate the performance of GRAPE/GARLIC [33], a recently described combination of algorithms that execute in silico retrobiosynthesis of nonribosomal peptide and polyketide natural products and comparative analysis of the resulting biosynthetic information. Our results represent, to our knowledge, the first comparative analysis of the ability of molecular similarity algorithms to quantify the chemical similarity of natural products, and suggest important roles for both circular fingerprints and retrobiosynthetic approaches in the targeted exploration of natural product chemical space and in genome mining for the identification of bioactive natural products.

## Results

Modular natural products can be characterized by a number of structural or biosynthetic features, including the nature of the enzymatic assembly line responsible for their biosynthesis (nonribosomal peptide, polyketide, or hybrid), their size, the presence or absence of starter units, their pattern of macrocyclization, and the action of tailoring reactions such as glycosylation, thiazole/oxazole formation, chlorination, or N-methylation. In order to evaluate the impact of each of these features in turn on chemical similarity search, we developed LEMONS (Library for the Enumeration of MOdular Natural Structures). LEMONS is a Java software package designed to enumerate hypothetical natural product structures given a user-determined set of biosynthetic parameters. Each hypothetical structure is subsequently modified by substituting one or more monomers, or by adding, removing, or changing the site of one or more tailoring reactions. The modified structure can then be compared to the entire library of original structures, using a two-dimensional fingerprint or another chemical similarity method. A correct match is scored if the modified structure displays greater chemical similarity to the original structure than to any of the other structures within the library. This process is repeated for each modified structure in turn, and the proportion of correct matches is determined for each chemical similarity method. LEMONS can thus be used to calculate the percentage of correct matches between original and modified structures for any chemical similarity method, using a user-input list of possible monomers and tailoring reactions.

**Fig. 2:**
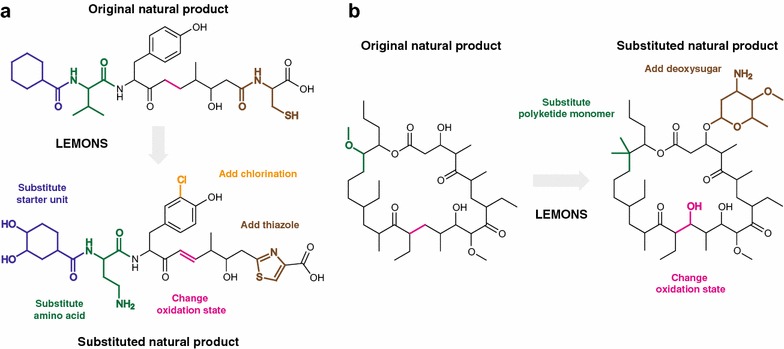
Examples of original and modified structures generated by LEMONS. **a** A linear hybrid nonribosomal peptide/polyketide natural product containing an alicyclic starter unit is derivatized by substitution of an amino acid, the starter unit, and a polyketide monomer, in addition to chlorination and heterocyclization/oxidation to form a thiazole. **b** A macrocyclic polyketide is derivatized by substitution of two polyketide monomers and glycosylation by the deoxysugar actinosamine

Our framework for the present study was as follows. First, we conduct a simple proof-of-concept validation of the LEMONS approach, by using LEMONS to generate libraries of short, linear proteinogenic peptides. Next, we use LEMONS to generate libraries of linear hypothetical nonribosomal peptides, polyketides, and hybrid natural products. We analyze the performance of each similarity method for each family of natural products, and consider the impact of monomer composition on similarity search. We also consider the impact of starter units, as are found in many modular natural product biosynthetic pathways. As the performance of some methods is claimed to exhibit a ligand size dependency, we next evaluate the relationship between natural product size (number of monomers) and similarity search. To more closely approximate the enzymatic tailoring reactions that occur in biosynthetic pathways, we then quantify the impact of macrocyclization and glycosylation. Finally, we use LEMONS to generate realistic libraries of extensively tailored natural products, such as those shown in Fig. 2, and provide general guidance for chemical similarity search in modular natural product chemical space.

As an initial proof-of-concept experiment, we used LEMONS to investigate the performance of chemical similarity methods given libraries of short polymers of proteinogenic amino acids. A library of 100 oligomers was generated, each with a length of 4–15 amino acids. A single amino acid was substituted within each structure, and the Tanimoto coefficient of the modified structure to each of the 100 original structures was calculated using 18 different chemical similarity methods (Table 1, Methods). This process was repeated by substituting an amino acid within each of the 100 original structures in turn, after which the number of correct matches between original and modified structures was determined. The entire experiment was repeated 100 times. Thus, a total of 10^4^ original structures and 10^4^ modified structures were generated, for a total of 10^6^ comparisons per similarity method (or 1.8 × 10^7^ in total per experiment).

**Table 1:** Chemical similarity methods evaluated in this study

Method	Type
ECFP0	Circular fingerprint
ECFP2	Circular fingerprint
ECFP4	Circular fingerprint
ECFP6	Circular fingerprint
FCFP0	Circular fingerprint
FCFP2	Circular fingerprint
FCFP4	Circular fingerprint
FCFP6	Circular fingerprint
MACCS	Substructure keys-based fingerprint
PubChem	Substructure keys-based fingerprint
E-State	Substructure keys-based fingerprint
Klekota–Roth	Substructure keys-based fingerprint
CDK (default)	Topological fingerprint
CDK (extended)	Topological fingerprint
CDK (hybridization)	Topological fingerprint
CDK (graph-only)	Topological fingerprint
LINGO	Lexicographic fingerprint
GRAPE/GARLIC	Retrobiosynthesis and alignment

**Fig. 3:**
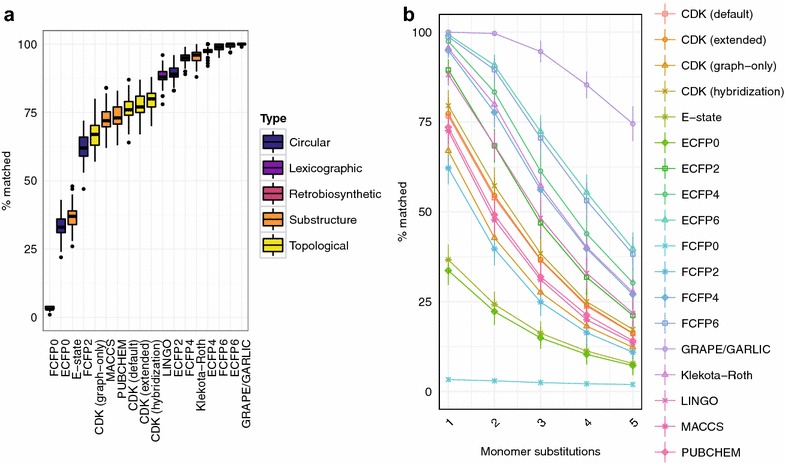
Chemical similarity method performance on hypothetical libraries of linear peptides. **a** Percentage of correct matches after substitution of a single proteinogenic amino acid in a library of hypothetical linear oligopeptides. **b** Trends in percentage of correct matches with substitution of one to five proteinogenic amino acids

Our results indicated that most chemical similarity algorithms performed reasonably well in this simple test (Fig. 3a). In general, circular and retrobiosynthetic algorithms performed best. It is unsurprising that the accuracy of GRAPE/GARLIC approached 100% (99.99% of structures correctly matched) because, in the absence of structural features such as macrocyclizations, tailoring reactions, or nonproteinogenic monomers, this method essentially performs a Needleman–Wunsch alignment of amino acid polymers given their chemical structures. Consequently, the performance of GRAPE/GARLIC was better than any two-dimensional fingerprint (one-sided Brunner–Munzel paired rank test, *P* ≤ 1.4 × 10^−14^ for all comparisons). A significant positive correlation between accuracy and radius was observed for circular fingerprints (Kendall’s *τ* = 0.85, *P* < 10^−300^). The correlation between accuracy and radius remained significant even with the ECFP0 and FCFP0 fingerprints removed from the analysis (*τ* = 0.78, *P* = 5.7 × 10^−300^) [34]. Significant variability in performance was also observed for substructure-based fingerprints. The Klekota–Roth fingerprint was particularly accurate (95.50%), outperforming several circular fingerprints (ECFP0, ECFP2, FCFP0, and FCFP2; one-sided Brunner–Munzel paired rank test, all *P* < 2.2 × 10^−16^), while the performance of the electrotopological state index fingerprint (E-state) was not statistically different from that of the ECFP0 fingerprint (36.67 and 33.66%, respectively; two-sided Brunner–Munzel paired rank test, *P* > 0.9999). Finally, the lexicographic fingerprint LINGO, which calculates molecular similarity based on the textual representations of two chemical structures as SMILES [35], has comparable accuracy to topological fingerprints given conventional benchmark data [36]. Its accuracy of 88.17% in this simple experiment suggests that it may be an acceptable method for similarity search in a large natural product-like chemical space when computational resources are very limited [37].

We repeated this proof-of-concept experiment four times, substituting two, three, four, and five proteinogenic amino acids in turn (Fig. 3b). Plotting the trend of correct matches against number of substituted monomers revealed that the performance of all methods decreased when a greater number of monomers were substituted. However, with a greater number of monomer substitutions, a more clear separation in accuracy between methods was observed. No change in the ranking of chemical similarity methods was observed as the number of monomer substitutions was increased.

**Fig. 4:**
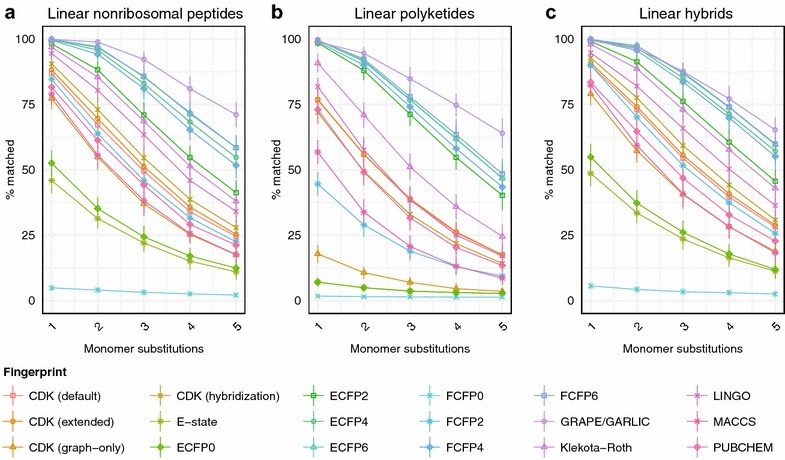
Chemical similarity method performance on hypothetical libraries of linear products. Trends in percentage of correct matches with substitution of one to five monomers within a hypothetical nonribosomal peptide (**a**), polyketide (**b**), or hybrid natural product (**c**)

We next sought to generate more realistic hypothetical natural product structures in order to better evaluate the performance of chemical similarity algorithms in natural product-like chemical space. In addition to proteinogenic amino acids, most peptidic natural products contain a diverse range of nonproteinogenic amino acids derived from primary metabolism by enzymatic tailoring reactions [38]. These building blocks significantly increase the structural diversity of peptide natural products, altering their physicochemical properties and introducing unique substructures or topologies. We therefore incorporated both proteinogenic amino acids and a set of 32 nonproteinogenic amino acids from bacterial and fungal nonribosomal peptides [39] to survey nonribosomal peptide chemical space (Additional file 1: Table S1). Type I polyketides are another large and pharmaceutically important class of modular natural products. In order to profile polyketide chemical space, we incorporated seven common polyketide monomers at all possible oxidation states into LEMONS scaffold generation (Additional file 1: Table S1). Finally, hybrid nonribosomal peptide-polyketide systems are responsible for the production of several valuable bioactive metabolites. We therefore generated linear hybrid polymers containing proteinogenic and nonproteinogenic amino acids and polyketide monomers. A comparison of algorithm performance on short linear polymers corresponding to hypothetical nonribosomal peptides, polyketides, and hybrid natural products is shown in Fig. 4 (see also Additional file 2: Fig. S1). Averaged across all fingerprints, similarity search with hypothetical nonribosomal peptides was 10.78% more accurate than with proteinogenic peptides (one-sided Brunner–Munzel test, *P* < 2.2 × 10^−16^). Similarity search was also more accurate for hypothetical nonribosomal peptides than for polyketides, by a margin of 13.07% (*P* < 2.2 × 10^−16^), 2.32% more accurate for hybrid natural products than nonribosomal peptides (*P* = 4.5 × 10^−8^), and 15.39% more accurate for hybrid natural products relative to polyketides (*P* < 2.2 × 10^−16^), averaged across all chemical similarity methods. These observations suggest that the unique properties of the nonproteinogenic amino acids found in bacterial and fungal nonribosomal peptides generally facilitate chemical similarity search.

In addition to nonproteinogenic amino acids and polyketide monomers, modular natural products commonly contain starter units that mediate biological activity, including short- and long-chain straight and branched fatty acids, aromatic and alicyclic acids, and amino acid derivatives [40]. These starter units might be expected to make a significant contribution to chemical similarity search due to their unique substructures or topologies. We investigated the impact of common starter units on similarity search in natural product-like chemical space by diversifying hypothetical hybrid natural products with 23 common starter units, including 4 fatty acids, 13 aromatic acids, 3 alicyclic acids, and 3 small starter units (Additional file 1: Table S1). In general, the performance of each similarity search method was similar for linear hybrid natural products with and without starter units (Additional file 3: Fig. S2). However, significant differences were observed in performance when comparing starter unit and non-starter unit substitution: for instance, the percentage of natural products correctly matched by the graph-only CDK fingerprint was 16.22% higher for starter unit substitutions, but 30.47% lower for the Pubchem fingerprint. Averaged across all fingerprints, a starter unit substitution was equivalent to 1.70 non-starter-unit substitutions (Additional file 3: Fig. S2). These results suggest that, in general, similarity search is strongly influenced by the unique structural properties of modular natural product starter units. While this phenomenon may facilitate the clustering of natural products of the same family that share a common starter unit, it may represent an obstacle to the cheminformatic identification of natural product families with variable starter units.

**Fig. 5:**
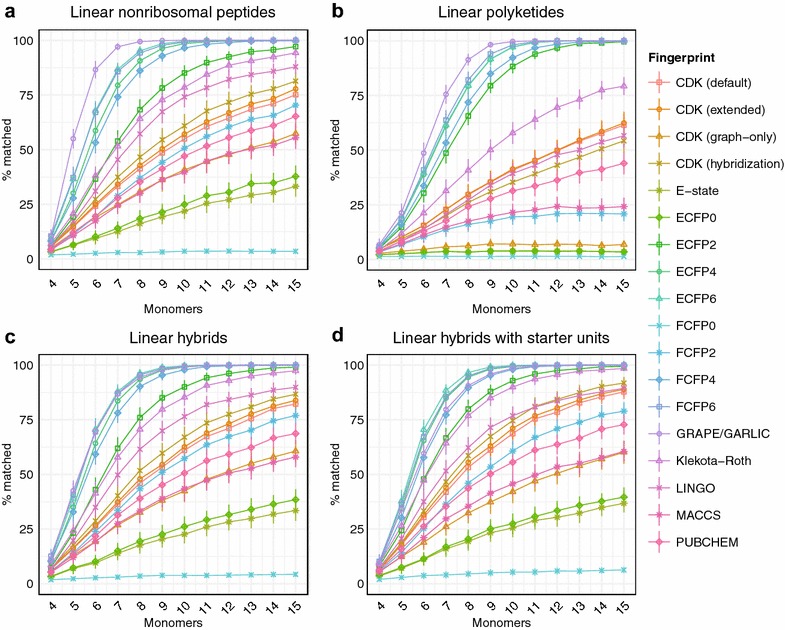
Chemical similarity method performance on hypothetical libraries of linear natural products as a function of natural product size. Trends in percentage of correct matches with substitution of three monomers within hypothetical linear nonribosomal peptides (**a**), polyketides (**b**), hybrid natural products (**c**), or hybrids with starter units (**d**) containing five to fourteen monomers

The performance of some fingerprints is known to exhibit a significant ligand size dependency [41]. Different chemical similarity search strategies may therefore exhibit optimal performance on compound families with lower or higher average molecular weights. We investigated the effect of size on chemical similarity algorithm performance by generating libraries of hypothetical natural products of fixed sizes. Libraries of hypothetical nonribosomal peptides, polyketides, hybrids, and hybrids with starter units consisting of 4 to 15 monomers were generated, and the performance of each algorithm with 3 monomer substitutions was evaluated (Fig. 5). In general, the rankings of chemical similarity methods by accuracy remained reasonably consistent regardless of natural product size, indicating that methods which perform well for smaller natural products tend to also perform well for larger natural products.

**Fig. 6:**
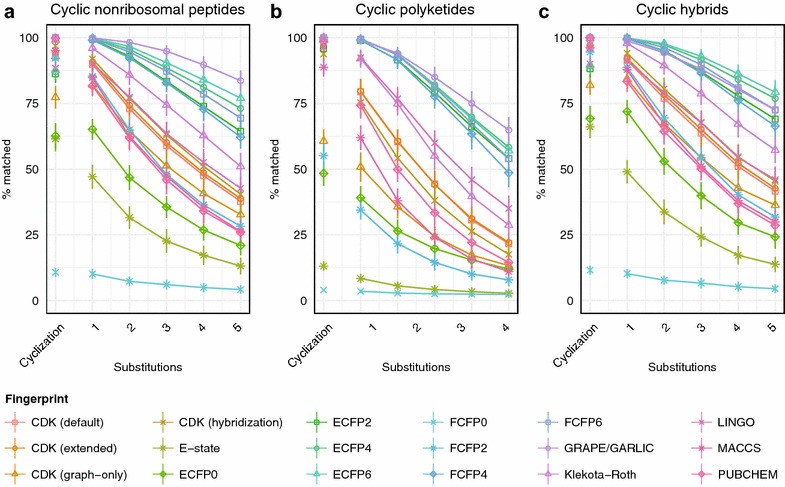
Chemical similarity method performance on hypothetical libraries of cyclic natural products. Trends in percentage of correct matches with substitution of one to five monomers or the site of macrocyclization within a hypothetical cyclic nonribosomal peptide (**a**), polyketide (**b**), or hybrid natural product (**c**)

Many natural products undergo regioselective macrolactone or macrolactam cyclizations, which are essential to their biological activity [42]. In order to evaluate the impact of macrocyclization on natural product similarity search, we generated cyclic and branched hypothetical nonribosomal peptides, polyketides, and hybrids (Fig. 6). Averaged across all fingerprints, substitution of the cyclization pattern was equivalent to substitution of 0.75 monomers in a hybrid natural product. Notably, ECFP fingerprints with a radius between 2 and 6 were at least 10% less accurate when the pattern of macrocyclization was changed relative to the substitution of a single monomer. These results indicate that macrocyclization makes a relatively small contribution to natural product similarity search, except in methods that heavily weight local atom environments.

**Fig. 7:**
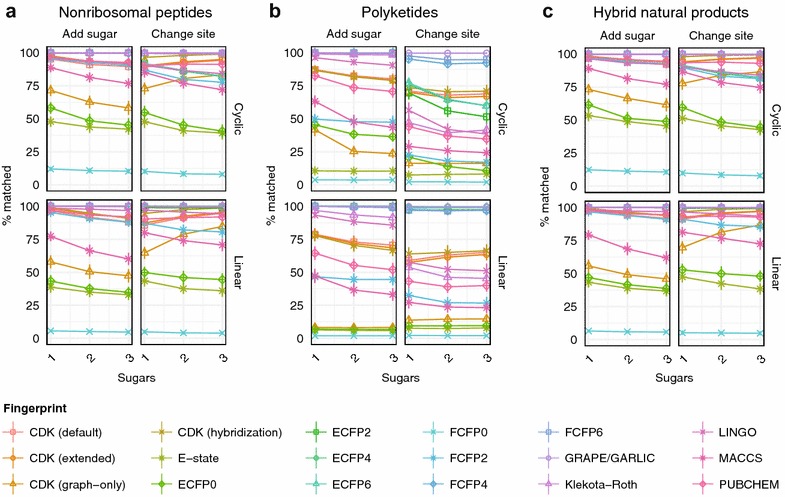
Chemical similarity method performance on hypothetical libraries of glycosylated natural products. Trends in percentage of correct matches with addition of one to three hexose or deoxsugars, or substitution of one to three glycosylation sites, within hypothetical linear or cyclic nonribosomal peptides (**a**), polyketides (**b**), or hybrid natural products (**c**)

During natural product biosynthesis, enormous structural diversity is created from a limited number of monomers by a diverse array of enzymatic tailoring reactions [20]. These reactions contribute significantly to the structural complexity of natural products. We modelled the effect of tailoring reactions on chemical similarity search by generating linear and cyclic nonribosomal peptides, polyketides, and hybrid natural products with one to three hexose or deoxysugars (Additional file 4: Table S2), and additionally analyzed the effect of changing the site of glycosylation (Fig. 7). For hybrid natural products, the magnitude of the effect of adding a sugar to an untailored scaffold was only slightly different from the effect of changing the site of glycosylation (mean accuracy for all fingerprints 83.86 vs. 83.36%, respectively), although the difference was highly significant (two-sided Brunner–Munzel paired-rank test, P < 2.2 × 10^−16^). Although no fingerprint was more accurate with the addition of three sugar moieties than with the addition of only one, the accuracy of all four topological fingerprints improved when the sites of three sugars were changed relative to only one (Brunner–Munzel test, *P* < 2.2 × 10^−16^). For linear hybrid natural products, the addition of one hexose or deoxysugar had an effect on accuracy equivalent to substituting 1.16 monomers, while changing the site of glycosylation was equivalent to substituting 1.17 monomers. These results suggest that, for non-topological fingerprints, the effect of diversification of modular natural products with hexose and deoxysugars is comparable to the substitution of an amino acid or ketide monomer.

**Fig. 8:**
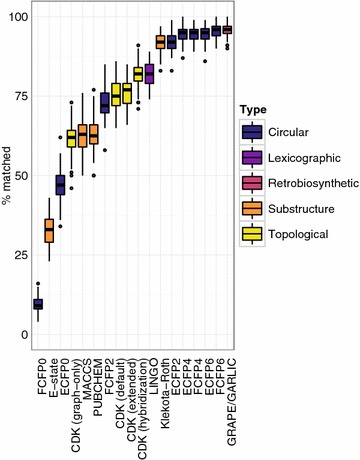
Chemical similarity method performance on hypothetical libraries of complex hybrid natural products. Percentage of correct matches after substitution of two monomers in hypothetical libraries of complex hybrid natural products with variable macrocyclization, glycosylation, heterocyclization, halogenation, and N-methylation

Having considered the impact of a number of biosynthetic features on chemical similarity search, we finally sought to quantify the performance of each chemical similarity method by generating realistic libraries of extensively tailored natural products, and thereby derive general recommendations for similarity search in modular natural product chemical space. We used LEMONS to generate libraries consisting in equal parts of cyclic and linear hybrid natural products with 4–15 monomers. Two monomers were substituted, and four tailoring reactions (glycosylation, N-methylation, halogenation, and oxazole/thiazole formation) were considered. Each type of reaction was executed on 50% of original structures, with a 25% chance of removing the tailoring from the modified structure and a 25% chance of changing the site of the reaction in the modified structure. As with proteinogenic amino acid oligomers, circular and retrobiosynthetic methods generally demonstrated the best performance for complex hypothetical natural products (Fig. 8). GRAPE/GARLIC and FCFP6 were equally accurate, i.e., did not give statistically different results, with mean accuracies of 95.80 and 95.53% of structures matched, respectively (Brunner–Munzel paired rank test, *P* = 0.42). The superior performance of the FCFP6 fingerprint to the ECFP6 fingerprint suggests that modular natural products may be enriched relative to small peptides in functional properties related to ligand binding (mean accuracy 95.53 and 94.67%, respectively; one-sided Brunner–Munzel paired rank test, *P* = 4.5 × 10^−5^). The positive correlation between circular fingerprint radius and accuracy was reproduced (Kendall’s *τ* = 0.69, *P* < 10^−300^) and remained significant with the ECFP0 and FCFP0 fingerprints removed from the analysis (*τ* = 0.48, *P* < 10^−300^). Among substructure-based fingerprints, the Klekota–Roth fingerprint was the most accurate, with accuracy not significantly different from the ECFP2 fingerprint (mean accuracy 91.77 and 91.99%, respectively; two-sided Brunner–Munzel paired rank test, *P* = 0.73). The relatively poor performance of the E–state, MACCS, and PubChem fingerprints suggests that the substructures indexed by these fingerprints are of limited use in chemical similarity search for modular natural products.

## Discussion

Modular natural products are attractive leads in drug discovery due to their evolved biological activities, but it is unclear which chemical similarity methods perform best in the chemical space occupied by their unique and complex scaffolds. Here, we present LEMONS, an algorithm for the enumeration of hypothetical natural product structures, and use it both to conduct a systematic comparison of chemical similarity methods for natural products, and to quantify of the impact of various structural parameters on similarity search. We find that, in general, size and macrocyclization have relatively little impact on similarity search, whereas the inclusion of nonproteinogenic amino acids and starter units more strongly affect similarity search. Circular fingerprints and a recently described retrobiosynthesis and alignment method consistently performed best across experimental conditions. There was a strong correlation between circular fingerprint radius and accuracy. Hashed topological fingerprints, which were outperformed by at least one fingerprint of every other type, appear to be an inappropriate strategy for natural product similarity search. Among substructure keys-based fingerprints, the Klekota–Roth fingerprint was most accurate. This fingerprint considers substructures which were determined to be privileged with respect to biological activity; it is conceivable that these substructures are overrepresented among natural products due to their evolved bioactivities. In general, our results support the use of circular fingerprints and retrobiosynthetic approaches for chemical similarity search in modular natural product chemical space.

**Fig. 9:**
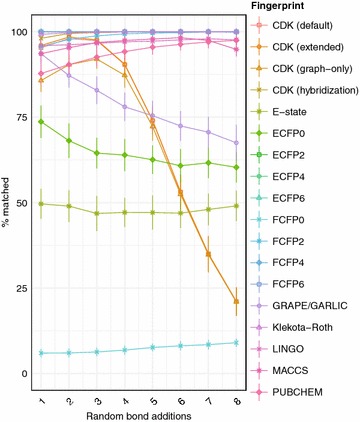
Effect of random bond addition to chemical similarity method performance. Trend in percentage of correct matches after substitution of a single monomer in hypothetical libraries of linear hybrid natural products with one to eight random bonds

An important limitation of the GRAPE/GARLIC method is its reliance on the assumption that biosynthetic logic can be codified in a rule-based manner, and applied to chemical structures to reconstruct their biosynthesis. However, natural product biosynthesis is diverse and incompletely understood, such that it is impossible to encode every possible tailoring reaction. In order to quantify the limitations of a retrobiosynthetic approach to similarity search, we added random bonds between any two atoms in natural product structures, simulating the effect of a tailoring reaction which violates the retrobiosynthetic logic built into the GRAPE algorithm (Fig. 9). We observed a monotonic decrease in the accuracy of GRAPE/GARLIC with the addition of random bonds between or within residues to hypothetical modular natural product structures, with each random bond associated with a 5.42% decrease in accuracy. The performance of GRAPE/GARLIC was equivalent to that of the average circular fingerprint with the addition of 2.99 random bonds, the average topological fingerprint with the addition of 4.91 random bonds, and the average substructure keys-based fingerprint with the addition of 2.80 random bonds. Our results suggest that although GRAPE/GARLIC generally outperforms conventional algorithms for calculating molecular similarity of natural products when retrobiosynthetic logic applies, its performance is likely to be poorer for classes of modular natural products whose biosynthesis is poorly understood, or which incorporates non-thiotemplated pathways. A further limitation of GRAPE/GARLIC is its relatively intensive use of computational resources in comparison to conventional fingerprints. Although the performance of retrobiosynthesis and alignment is heavily dependent on the types of compounds subjected to analysis, in most cases GRAPE/GARLIC is more computationally intensive than two-dimensional fingerprints. Efforts are currently underway to improve the performance of GRAPE/GARLIC in a future release.

As an extensible, open-source method to enumerate hypothetical natural product structures, LEMONS has diverse potential applications in bioinformatics beyond analysis of molecular similarity algorithms. Several reports, for instance, have highlighted the applicability of targeted hypothetical structure enumeration to the discovery of natural products from tandem mass spectrometry data of complex bacterial extracts [43, 44]. However, these methods require varying degrees of manual intervention to produce libraries of hypothetical structures. In contrast, LEMONS can be leveraged as a platform for rapid, untargeted exploration of desired regions of chemical space or natural product families. For instance, ‘macrolide-like’ chemical space could be explored by generating cyclic polyketides containing seven or eight monomers, with zero, one, or two glycosylations. The integration of untargeted hypothetical structure enumeration via LEMONS with existing methods for locating hypothetical chemical structures within mass spectral data may facilitate genome mining for desired secondary metabolites. Alternatively, the algorithm can be expanded to profile chemical space more broadly, particularly as its extensibility facilitates the addition of any number of new monomers or tailoring reactions. The ability to randomly profile a particular region of natural product chemical space may provide insights into the chemical evolution of natural product families.

A limitation of LEMONS with respect to the broader applications of hypothetical natural product structure enumeration is that its design considers only linear or cyclic permutations of monomers. Consequently, in its present implementation, the algorithm is only extensible to biosynthetic classes that can be modelled as post-translationally modified linear or cyclic polymers. Thus, while LEMONS could be extended to profile natural product classes such as ribosomally synthesized and posttranslationally modified peptides (RiPPs), a graph-based method of structure enumeration would be required to model classes such as terpenes or aminoglycosides, in which monomers (isoprene units or sugars, respectively) may more appropriately be considered subgraphs which can potentially connect to other subgraphs at multiple sites.

## Conclusions

We describe LEMONS, an open-source and easily extensible algorithm for untargeted enumeration of modular natural product chemical structures. We use this algorithm to benchmark chemical similarity methods for modular natural products, finding that circular fingerprints and a newly described retrobiosynthetic approach (GRAPE/GARLIC) perform best, whereas topological fingerprints and most substructure-based fingerprints perform less well (with the notable exception of the Klekota–Roth fingerprint). Additionally, we investigate the impact of biosynthetic parameters on similarity search, finding that size and macrocyclization have relatively little impact on similarity search, whereas inclusion of nonproteinogenic amino acids and starter units have a stronger effect. Our results lead us to recommend the use of circular fingerprints and retrobiosynthetic approaches for modular natural product similarity search, and our method has diverse potential applications in chemical space exploration and microbial genome mining.

## Methods

### Development of an algorithm to enumerate hypothetical natural product structures

We developed LEMONS (Library for the Enumeration of MOdular Natural Structures), a Java software package designed to investigate the performance of chemical similarity metrics by enumerating hypothetical natural product structures given a user-determined set of biosynthetic parameters. LEMONS first enumerates a library of hypothetical natural product structures given a list of possible monomers and tailoring reactions. Each hypothetical natural product structure may subsequently be modified by substituting one or more monomers, or by adding, removing, or changing the site of one or more tailoring reactions. The modified structure is compared to the entire library of original structures using a two-dimensional molecular fingerprint, and the rank of the correct match (i.e., of the modified structure to the original structure from which it was derived) is determined. This process is repeated for each hypothetical structure within the library, and the rank distribution of each molecular fingerprint is written to a file. LEMONS uses the Chemistry Development Kit (version 1.5.9) for chemical structure generation and molecular fingerprint calculation [45]. LEMONS source code is available at http://github.com/magarveylab/lemons.

### Linear structure generation

The first step in the LEMONS algorithm is the enumeration of a library of linear hypothetical natural product structures. A permutation of monomers is selected at random from one or more monomer sets in order to generate a polymer of a given size range. By default, four possible sets of monomers are included within LEMONS, including proteinogenic and nonproteinogenic amino acids, polyketide monomers, and starter units. LEMONS includes 45 nonproteinogenic amino acids derived from fungal and bacterial nonribosomal peptides, including β-hydroxylated and β-methylated amino acids, α-keto acids, β-amino acids, and other commonly occurring modified amino acids (Additional file 1: Table S1). LEMONS also includes 26 polyketide monomers, corresponding to 7 common polyketide extender units (malonate, methylmalonate, ethylmalonate, methoxymalonate, propionate, isobutyrate, and 2-methylbutyrate) at all possible oxidation states (Additional file 1: Table S1). Finally, LEMONS includes 23 common starter units, including four fatty acids, thirteen aromatic starter units, three alicyclic starter units, and three small starter units (Additional file 1: Table S1). A template is provided within the LEMONS source code to facilitate the addition of new monomer sets to the software package.

### Tailoring reaction detection and execution

The second step in the LEMONS algorithm is the detection of all possible tailoring reactions. Polymers are converted to chemical structures, and potential sites of each reaction on the resulting chemical structure are identified. Tailoring reactions to be executed on the original library of hypothetical natural product structures are set via the ‘–initial_reactions’ option of the command line interface in a probabilistic manner, such that the argument ‘halogenation 0.5’ will cause LEMONS to attempt to execute a halogenation reaction on 50% of the scaffolds, while the arguments ‘halogenation 1’ and ‘halogenation 2’ will cause LEMONS to attempt to execute one and two halogenation reactions, respectively, on each scaffold. Five possible tailoring reactions are included in LEMONS by default (cyclization, halogenation, glycosylation, thiazole/oxazole formation, and amino acid N-methylation). A template is provided within the LEMONS source code to facilitate the addition of new reactions to the software package.

The cyclization reaction within LEMONS creates cyclic or branched hypothetical natural product structures by cyclizing either an N-terminal amine or a free hydroxyl at the C-terminal carboxylic acid. Only sp^3^ carbon-bound hydroxyls are considered potential sites of cyclization, permitting cyclization on serine and threonine residues or β-hydroxylated amino acids. The halogen reaction executes chlorination or bromination with equal probability. In order to capture the diverse patterns of natural product halogenation [46], any non-backbone carbon is considered a possible site of halogenation. The glycosylation reaction leverages a library of 69 deoxy and hexose sugars [39] to generate O-glycosylated hypothetical natural product structures (Additional file 4: Table S2). Both sp^2^ and sp^3^ carbon-bound hydroxyls are considered potential sites of O-glycosylation with a randomly selected sugar moiety. The thiazole and oxazole-forming reaction considers all cysteine and serine residues, respectively, as potential sites of thiazole or oxazole formation, and allows for bis-thiazole or oxazole formation. The N-methylation reaction considers all backbone amide nitrogens derived from proteinogenic and nonproteinogenic amino acids as potential sites of N-methylation.

### Generation of modified structures

Once all potential tailoring reactions have been detected and a subset selected for each structure in the library of hypothetical natural product structures, the original structure is modified by substituting one or more monomers, or by adding, removing, or changing the site of one or more tailoring reactions, in order to produce a derivative of the same in silico assembly line. The set of monomers substituted into the original polymer can be specified independently from the set of monomers used to construct the original polymer. If the substitution set includes starter units, these monomers will only be substituted at the first residue. In order to maximize chemical diversity, each monomer in the original scaffold will be substituted before an individual monomer is swapped twice. Monomers which are the sites of chemical tailoring reactions are not substituted. The command line interface additionally allows the independent specification of new tailoring reactions to add to the original scaffold, tailoring reactions to remove from the original scaffold, and tailoring reactions whose sites may be changed. Thus, for instance, an unmethylated peptide can be methylated, a methylated peptide can have its N-methyl group removed, and the site of N-methylation can be varied. The execution of the modified reaction set allows for the conversion of the resulting modified structure to a chemical scaffold.

### Comparison of modified and original structures

The final step in the LEMONS algorithm compares the ability of seventeen molecular fingerprints (Table 1) to correctly match the original and modified hypothetical natural product structures. The Tanimoto coefficient of a modified structure to each structure in the original library is calculated, and the rank of the Tanimoto coefficient to the original structure is calculated. This process is repeated for every structure within the original library, and the rank distribution of the correct match is written to a file.

### Generation of chemical fingerprints

Three predominant approaches exist for computing two-dimensional molecular fingerprints [4]. The first, substructure keys-based fingerprints, set the bits within a molecular fingerprint bit sequence based on the presence or absence of predefined structural features. The MACCS (Molecular ACCess System) fingerprint is among the best-known substructure keys-based fingerprints [47]. The PubChem database implements another substructure keys-based fingerprint for similarity search, based on 881 structural keys [48]. Both the 79 electrotopological state (E-state) fragments defined by Hall and Kier [49] and the 4860 unique chemical substructures which enrich for biological activity defined by Klekota and Roth [50] have also been used within substructure keys-based fingerprints implemented in the Chemistry Development Kit [45].

Topological or path-based fingerprints describe fragments of a molecule, typically by enumerating all paths through a molecule up to a certain number of bonds, then hashing each path to create a fingerprint. Topological fingerprints typically encode information about the atom types and number and type of bonds within each path. While individual bits correspond to unique structural features in substructure-based fingerprints, a single bit may correspond to several features in topological fingerprints, a phenomenon known as bit collision. The Daylight fingerprint [51] is probably the best-known topological fingerprint. Variants of the Daylight fingerprint are implemented in several open-source cheminformatic libraries, including RDKit [52] and the Chemistry Development Kit (CDK) [45].

Circular fingerprints are also hashed fingerprints which encode circular atom environments up to a maximum bond radius from the central atom rather than looking for paths through a chemical graph. Extended-connectivity fingerprints (ECFPs) [53] encode properties including chemical element, number of heavy-atom neighbours, number of hydrogens, and isotope and ring information, whereas functional connectivity fingerprints (FCFPs) [53] encode pharmacophoric features related to ligand-binding. The maximum bond length radius is appended to the end of the name: thus the ECFP6 fingerprint is an extended-connectivity fingerprint with a diameter of 6 bonds.

Other fingerprints adopt lexicographic approaches to the generation of molecular fingerprints. LINGO [35], for instance, is a text-based fingerprint calculated based on canonical SMILES. Seventeen molecular fingerprints were evaluated in this study, including ECFP and FCFP circular fingerprints with radii of 0, 2, 4, and 6; four substructure keys-based fingerprints (MACCS, PubChem, E-state, Klekota-Roth); one lexicographic fingerprint (LINGO); and four Daylight-like topological fingerprints (Table 1). All molecular fingerprints were generated with the Chemistry Development Kit (CDK) [45], version 1.5.9.

### Statistical analysis

For each comparison, we reported the *P* value for either the Brunner–Munzel paired rank test or the Brunner–Munzel independence test. When utilizing a statistical hypothesis test, it is common practice to compare a *P* value to a pre-established significance level, e.g., *α* = 0.05. In some instances, we are performing 17 comparisons, and so would need to adjust the significance level for each individual comparison to account for the multiple comparisons. This could be done, for example, using a Bonferroni correction so that if we desired that the family wise error rate should not exceed 5%, then the significance level for each individual comparison would be 0.05/17 = 0.0029. We mention this only for completeness because, in the multiple testing scenarios we consider herein, the *P* values for the individual comparisons are so small that adjusting for multiple comparisons will not affect the conclusions presented.

## Additional files

**Additional file 1: Table S1.** Monomers used to construct libraries of hypothetical modular natural products in LEMONS.

**Additional file 2: Fig. S1.** Performance of individual chemical similarity methods on hypothetical libraries of linear peptides.

**Additional file 3: Fig. S2.** Chemical similarity method performance on hypothetical libraries of linear hybrid natural products with and without starter units. (*A*) Trends in percentage of correct matches after substitution of a single monomer in a library of hypothetical linear hybrid natural products with starter units. (*B*) Percentage of correct matches with substitution of the starter unit or one to five non-starter unit monomers.

**Additional file 4: Table S2.** Deoxy and hexose sugars used to tailor libraries of hypothetical modular natural products in LEMONS.
