# Supplementary figures and images for: Comparative analysis of chemical similarity methods for modular natural products with a hypothetical structure enumeration algorithm
Source: J Cheminform. 2017 Aug 16;9:46. doi: 10.1186/s13321-017-0234-y (PMC5559407; doi:10.1186/s13321-017-0234-y)

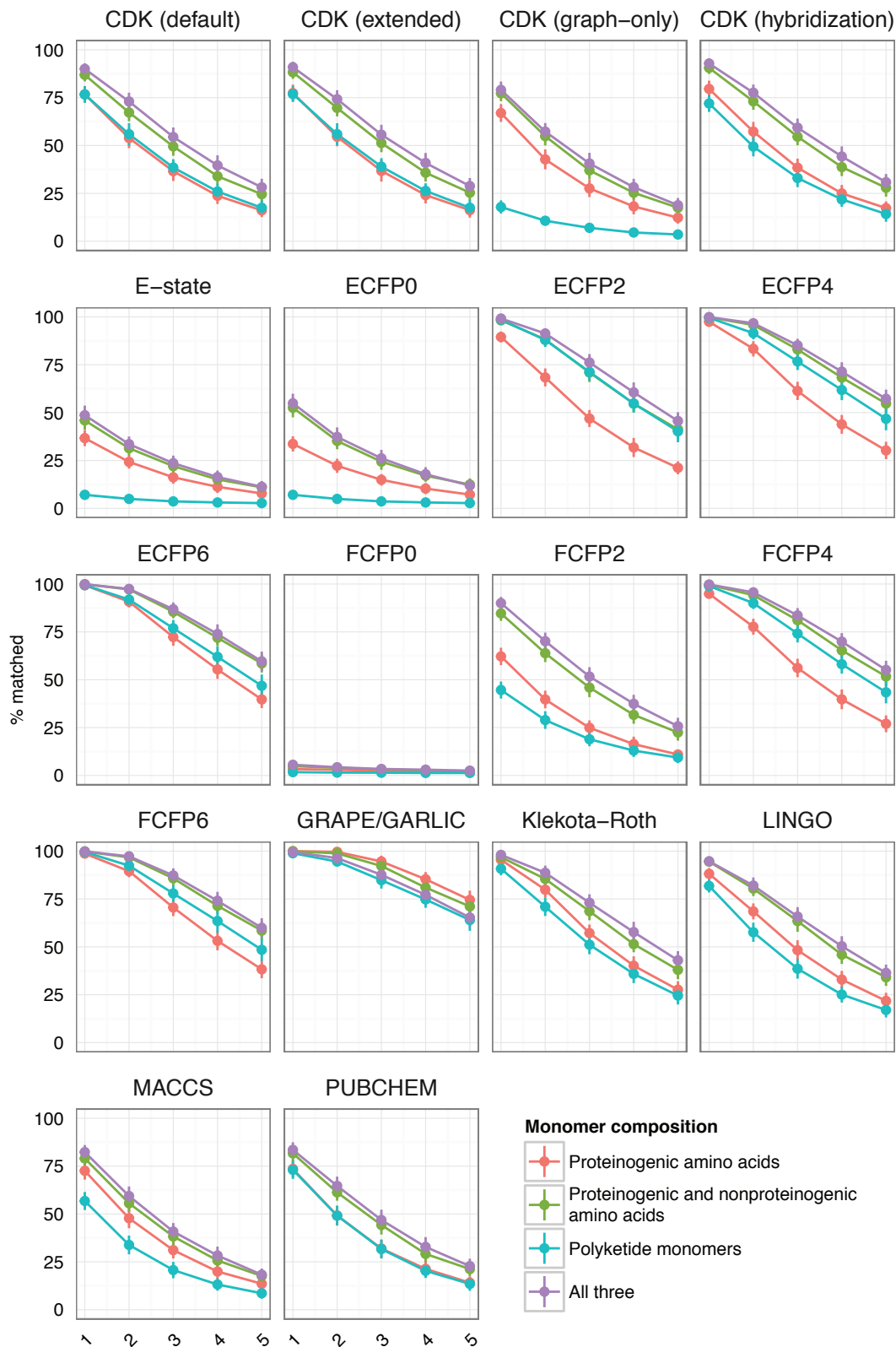

Supplement: Supplementary file 2 — Additional file 2: Fig. S1. Performance of individual chemical similarity methods on hypothetical libraries of linear peptides. [file 13321_2017_234_MOESM2_ESM.pdf]
