# Supplementary material for: Comparative analysis of chemical similarity methods for modular natural products with a hypothetical structure enumeration algorithm
Source: J Cheminform. 2017 Aug 16;9:46. doi: 10.1186/s13321-017-0234-y (PMC5559407; doi:10.1186/s13321-017-0234-y)
Supplement: Supplementary file 3 — Additional file 3: Fig. S2. Chemical similarity method performance on hypothetical libraries of linear hybrid natural products with and without starter units. (A) Trends in percentage of correct matches after substitution of a single monomer in a library of hypothetical linear hybrid natural products with starter units. (B) Percentage of correct matches with substitution of the starter unit or one to five non-starter unit monomers. [file 13321_2017_234_MOESM3_ESM.pdf]

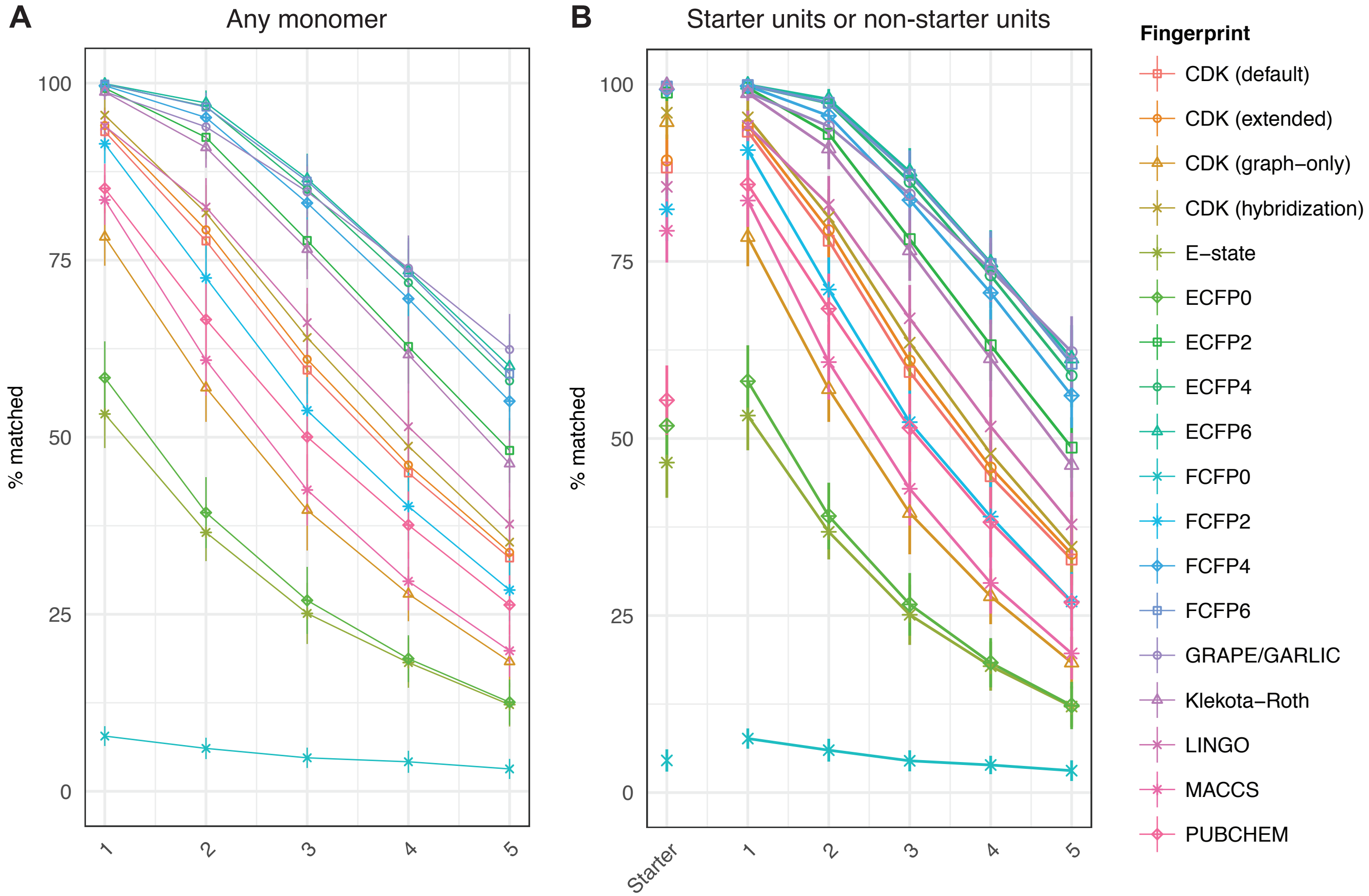
A
Any monomer
B
Starter units or non-starter units
% matched
100
75
50
25
0
1
2
3
4
5
Starter
Fingerprint
CDK (default)
CDK (extended)
CDK (graph−only)
CDK (hybridization)
E−state
ECFP0
ECFP2
ECFP4
ECFP6
FCFP0
FCFP2
FCFP4
FCFP6
GRAPE/GARLIC
Klekota−Roth
LINGO
MACCS
PUBCHEM
